# Developing Three-Dimensional (3D) Skin Models to Study the Interaction Between the Skin Microbiome and Immune Cells in Chronic Wounds

**DOI:** 10.7759/cureus.113304

**Published:** 2026-07-24

**Authors:** Laith Basch, Asha Gopu, Hannah Welp, Mahnoor Mukarram, Precious Ochuwa Imokhai, Kim Huynh, Shantanu Amin, Randy Jacobs

**Affiliations:** 1 Dermatology, Arizona College of Osteopathic Medicine, Midwestern University, Glendale, USA; 2 Dermatology, Medical College of Georgia, Augusta University, Augusta, USA; 3 Medicine, DeBusk College of Osteopathic Medicine, Lincoln Memorial University, Harrogate, USA; 4 Dermatology, Larkin Community Hospital Palm Springs Campus, Hialeah, USA; 5 Medicine, Montana College of Osteopathic Medicine, Rocky Vista University, Billings, USA; 6 Medicine, Arizona College of Osteopathic Medicine, Midwestern University, Glendale, USA; 7 Medicine, Kansas City University, Joplin, USA; 8 Dermatology, University of California (UCR) School of Medicine, Riverside, USA

**Keywords:** chronic wounds, delayed wound healing, skin microbiome, three-dimensional models, ­wound healing

## Abstract

Developing three-dimensional (3D) skin models provides a physiologically relevant platform to investigate the dynamic interactions between the skin microbiome and immune cells in chronic wounds, offering insights into delayed healing, persistent inflammation, and microbial dysbiosis. Traditional in vitro models fail to replicate the complex architecture of human skin and its immune-microbiome crosstalk, motivating the development of biomimetic systems that incorporate stratified epidermal layers, dermal fibroblasts, and innate immune components such as macrophages and neutrophils. Engineered 3D skin constructs inoculated with wound-associated microbiota, including *Staphylococcus aureus*, *Pseudomonas aeruginosa*, and *Cutibacterium acnes*, can reveal how biofilm formation, quorum sensing, and microbial metabolites influence immune cell recruitment and cytokine secretion. Dysregulated immune responses within these models, characterized by excessive interleukin-1 beta, tumor necrosis factor alpha, and reactive oxygen species production, mirror aspects of the inflammatory milieu observed in nonhealing wounds and highlight candidate pathways for therapeutic intervention. The incorporation of patient-derived immune cells into 3D co-culture systems further enables personalized modeling of wound-healing deficiencies and antimicrobial-resistance patterns. Applications of 3D skin models extend to the development of antibiofilm agents, immune-modulating therapies, and microbiome-targeted interventions. It is important to emphasize that the current evidence base is predominantly preclinical and in vitro; accordingly, these platforms should be viewed as a promising foundation for advancing precision-medicine approaches to chronic wound management, contingent on rigorous cross-laboratory and clinical validation.

## Introduction and background

The classification of wounds as acute or chronic depends primarily on the duration and trajectory of healing. By convention, a wound that fails to progress in an orderly and timely fashion through the normal phases of repair, or that does not achieve sustained anatomical and functional integrity within approximately 4-12 weeks (most commonly cited as four weeks), is designated chronic; the precise threshold varies with wound etiology and the source consulted, and we adopt this timeframe throughout for clarity. This distinction holds critical clinical importance, particularly given the growing burden that chronic wounds impose on modern healthcare systems. The rise in chronic wound prevalence stems from its association with common comorbid conditions, including diabetes, peripheral artery disease, and the aging process [1,2]. Chronic wounds present significant challenges to global healthcare systems, affecting approximately 10.5 million Medicare beneficiaries in the United States alone [3]. The financial burden is substantial: annual Medicare expenditure for wound care in both inpatient and outpatient settings has been estimated to fall between $28.1 billion and $96.8 billion [3]. This wide range reflects differing cost-estimation methods and case definitions rather than genuine uncertainty of that magnitude, and it should be interpreted accordingly. The five-year mortality of diabetic foot ulcers is comparable to that of several common cancers (approximately 30.5% vs. 31%, respectively) and has remained relatively stable since 2007 [4]; these are five-year figures rather than crude in-hospital mortality. Relative to this clinical and economic burden, chronic wound research attracts comparatively limited dedicated funding, drawing attention to a potential gap between disease burden and research allocation. This discrepancy makes it imperative to investigate the mechanisms underlying chronic wound pathogenesis.

To appreciate the need for novel interventions, it is first necessary to understand the complex pathophysiology of chronic wounds. Chronic wound healing is disrupted by a network of interacting processes involving persistent, dysfunctional inflammatory signaling, microbial colonization, and impaired tissue repair. These microenvironments feature excessive levels of proinflammatory mediators, including interleukin (IL)-1 beta, tumor necrosis factor alpha (TNF-α), and reactive oxygen species (ROS), which disrupt normal healing [5]. These mediators sustain an inflammatory state that blocks progression to the proliferative and remodeling phases. Chronic wounds also create favorable conditions for diverse microbial species, including *Staphylococcus aureus*, *Pseudomonas aeruginosa*, and *Cutibacterium acnes*, which form protective biofilms that evade host immunity and antibiotic treatment, further complicating healing [5]. Owing to this multifactorial nature, chronic wounds present significant management challenges. Clinically, this complexity translates into prolonged healing times, repeated outpatient visits, recurrent infection and hospitalization, elevated amputation risk, and diminished quality of life, and it helps explain why single-target therapies frequently fail. These clinical realities underscore the need for experimental platforms that capture several disease drivers simultaneously and that can diagnose and treat dysfunctional healing at its source.

**Figure 1:**
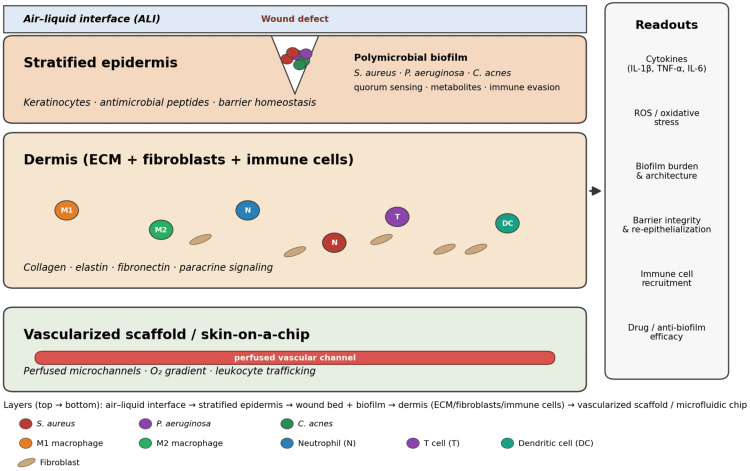
Schematic of an idealized 3D skin model for studying chronic wounds. The construct layers an air-liquid interface, a stratified epidermis (keratinocytes, antimicrobial peptides), and a wound bed harboring a polymicrobial biofilm (S. aureus, P. aeruginosa, C. acnes) that engages in quorum sensing, metabolite release, and immune evasion, a dermal compartment (ECM, fibroblasts, and innate/adaptive immune cells including M1/M2 macrophages, neutrophils, T cells, and dendritic cells), and a vascularized scaffold or microfluidic skin-on-a-chip providing perfusion, oxygen gradients, and leukocyte trafficking. Representative readouts are listed at right 3D: three-dimensional; ECM: extracellular matrix; IL-1β: interleukin-1 beta; IL-6: interleukin 6; TNF-α: tumor necrosis factor alpha; ROS: reactive oxygen species; DC: dendritic cell Image credit: This is an original image created by the author Laith Basch using Claude (Python code using Matplotlib; Anthropic, San Francisco, CA)

Traditional approaches to studying skin-microbiome-immune interactions include in vitro monolayer cell cultures, which have been widely used to observe cellular responses in host-microbial interactions [6]. Simple microbiome models have provided a platform for exploring bacterial interactions with monolayers of host cells, as demonstrated by *S. aureus*-induced serine protease activity and *Staphylococcus epidermidis*-triggered keratinocyte defense activation [7,8]. However, these two-dimensional (2D) models fail to capture the complexity of the human skin microenvironment, including its 3D architecture, extracellular matrix (ECM) interactions, and dynamic host-microbe interactions, making them poorly predictive [9]. It is useful to distinguish several classes of skin model that are often grouped together: 1) 2D monolayer cultures of a single cell type; 2) organotypic, reconstructed 3D equivalents such as reconstructed human epidermis (RHE) and full-thickness skin equivalents that recapitulate a stratified epidermis on a fibroblast-populated dermal compartment; 3) 3D bioprinted constructs, in which cells are deposited in defined spatial patterns; and 4) microfluidic “skin-on-a-chip” systems that add perfusion, mechanical cues, and controllable oxygen gradients. These classes differ substantially in architecture, immune and vascular integration, throughput, and cost, as summarized in Table 1 and depicted schematically in Figure 1. Chen et al. illustrated the practical consequences of this difference by comparing silver-nanoparticle treatment in 2D monolayer keratinocyte cultures and a 3D epidermal model: the 2D cultures showed inflammation-related cytotoxicity and oxidative stress at doses that produced no such effects in the 3D model [10]. Integrating these more advanced models into wound research, therefore, allows for more predictive insights into therapeutic outcomes.

**Table 1:** Comparison of the principal in vitro skin model classes used in chronic wound research 2D: two-dimensional; 3D: three-dimensional; ECM: extracellular matrix

Model class	Key features	Immune and vascular integration	Main strengths	Main limitations
2D monolayer culture	Single cell type on flat substrate; no stratification or ECM	None (typically single cell type)	Simple, low-cost, high throughput; widely used for cellular-response studies	Poor architecture; low predictivity; overestimates cytotoxicity
Reconstructed human epidermis	Stratified, differentiated epidermis at air-liquid interface	Limited; usually no vasculature; immune cells optional	Physiologic barrier; suited to topical drug and microbe studies	Short lifespan; lacks dermis/adnexa; higher cost
Full-thickness skin equivalent	Epidermis plus fibroblast-populated dermal compartment	Immune cells can be embedded; usually avascular	Recapitulates epidermal-dermal signaling; supports wounding	No perfusion; batch variability; standardization gaps
3D bioprinted construct	Cells/bioinks deposited in defined spatial patterns	Can pattern immune cells; vasculature emerging	Reproducible geometry; scalable; can include adnexa/neurovasculature	Requires bioink optimization; higher cost
Microfluidic skin-on-a-chip	Perfused microchannels with mechanical and O₂ control	Enables perfusion, leukocyte trafficking, and controllable gradients	Models systemic/dynamic responses and drug delivery	Lower throughput; limited standardization

The development of 3D human skin models has advanced wound-healing research by enabling more dynamic analysis of cutaneous physiology. These models more closely replicate the skin’s architecture by incorporating a native ECM and supporting paracrine signaling that more closely resembles that of native tissue [11]. This is achieved using recent advances in 3D bioprinting, which deposit bioinks into predesigned shapes and enable precise positioning of cells, thereby creating complex tissues that mimic native function [5,12]. Bioprinting produces structurally stable constructs that replicate tissue microenvironments while supporting living tissue through their metabolic and physiologic requirements [13]. Recent advances have enabled the integration of immune cells and wound-associated microbes into 3D skin models, enhancing their ability to replicate the native skin environment. Loomis et al. demonstrated that adding mixed skin microbiomes to 3D skin models produced significant changes in epidermal thickness, gene expression, and cell proliferation [14]. Understanding chronic wound pathophysiology and developing therapeutic interventions require in vitro replication of key processes in 3D constructs to study quorum sensing, biofilm formation, and microbial metabolite release. The primary aim of this narrative review is to examine the potential of 3D skin models as platforms for investigating the interactions between the skin microbiome and immune cells in chronic wounds, while highlighting emerging strategies, including antibiofilm agents, immune-modulating therapies, and microbiome-targeted interventions, that may eventually support precision-medicine approaches to chronic wound management.

Methods

This article is a narrative review and is reported in accordance with the Scale for the Assessment of Narrative Review Articles guidance for nonsystematic reviews [15]; it is not a systematic review and does not follow Preferred Reporting Items for Systematic reviews and Meta-Analyses reporting or include a formal risk-of-bias assessment or quantitative synthesis. To improve transparency and reproducibility, we disclose our search approach. We searched PubMed/MEDLINE, Scopus, and Web of Science for English-language articles, with an emphasis on work published between 2015 and 2026 and priority given to studies from 2020 onward; foundational earlier references were retained where they remain authoritative. Search terms were combined using Boolean operators and included variations and combinations of: “3D skin model”, “reconstructed human epidermis”, “full-thickness skin equivalent”, “bioprinted skin”, “skin-on-a-chip”, “skin microbiome”, “biofilm”, “chronic wound”, “diabetic foot ulcer”, “immune cells”, “macrophage”, “neutrophil”, “cytokine”, “*Staphylococcus aureus*”, “*Pseudomonas aeruginosa*”, and “*Cutibacterium acnes*”. We included primary studies and reviews addressing 3D skin or wound models, skin microbiome-immune interactions, or chronic wound pathophysiology, and excluded articles not available in English, conference abstracts without full data, and studies without relevance to cutaneous or wound biology. Reference lists of key articles were screened for additional sources. Because study designs, model systems, and outcome measures were heterogeneous, evidence was synthesized narratively, with greater weight given to peer-reviewed primary studies using human or human-derived 3D constructs. We note that at least one cited source is a preprint [16] and should be interpreted with appropriate caution pending peer review.

## Review

Pathophysiology of chronic wounds

The development of chronic wounds involves several interrupted healing phases that combine prolonged inflammation, microbial imbalance, and weakened immune responses [17]. Healing becomes deregulated by excessive proinflammatory cytokines and oxidative stress, which impair tissue regeneration and delay entry into the proliferative phase [18]. *S. aureus* and *P. aeruginosa* act as opportunistic, biofilm-forming pathogens that establish persistent infection in chronic wounds [19]. These organisms produce protective matrices that shield them from antimicrobial agents and immune surveillance while sustaining a cycle of unresolved inflammation and tissue deterioration [20]. This cycle prolongs the inflammatory phase and prevents progression through the normal stages of wound healing, thereby contributing to chronicity.

The risk of chronic wound development is highest among patients with diabetes mellitus, advanced age, immunosuppression, peripheral vascular disease, or restricted mobility [21]. In diabetic patients, bacterial colonization is favored by neuropathy and microvascular disease combined with impaired immune function [22]. Immune senescence associated with aging, together with a weakened skin barrier, makes older adults more susceptible to inflammatory dysregulation and microbial imbalance [18]. Patients receiving cancer therapy, autoimmune disease management, or immunosuppressive medications often experience poor microbial clearance and persistent infection [17]. Microbial communities form biofilms efficiently in tissues affected by venous stasis, arterial insufficiency, or pressure injury [20]. Together, these host vulnerabilities and local tissue conditions create an environment that favors chronic wound persistence.

A continuous feedback loop between immune dysfunction and microbial dysregulation allows chronic wounds to persist [21]. The combination of slow healing and infection with resistant microorganisms predisposes to severe complications such as sepsis and osteomyelitis [23]. Signs of infection in diabetic and immunocompromised patients are frequently difficult to detect because typical clinical features may be absent [19]. Nonhealing wounds also carry a risk of progression to systemic conditions such as bacteremia or endocarditis, particularly in individuals with compromised immunity [19]. Older adults experience worse morbidity because of treatment-related complications, including antibiotic resistance, adverse drug reactions, and pain from debridement [24], and multimorbidity slows healing while increasing the likelihood of chronicity [25]. This complexity makes clinical management difficult and underscores the urgency of interventions that target both the host immune response and the wound microbiota [18].

Immune-microbiome interactions are a central and evolutionarily conserved determinant of tissue homeostasis. Under normal conditions, the resident microbiota helps calibrate innate and adaptive immunity, promoting balanced defense, tolerance, and repair; when this balance is disrupted, dysbiosis can develop. Dysbiosis is characterized by an altered, often overgrown microbial community that paradoxically coincides with impaired local immune function. The microbiota influences immune cell development and function in part through microbial metabolites; short-chain fatty acids, for example, modulate the differentiation and activity of immune cells [26]. In the setting of chronic inflammation, this bidirectional signaling between the microbiota and the immune system is amplified and becomes particularly relevant to the management of chronic wounds, autoimmune conditions, and systemic inflammation. Understanding these interactions supports a therapeutic strategy based on ecological restoration of the microbiome to modulate immunity, rather than on microbial eradication alone. Notably, in vivo studies indicate that biofilm is present in the majority of chronic wounds; a meta-analysis by Malone et al. estimated a prevalence of approximately 78%, and earlier work by Bjarnsholt et al. proposed that persistent wound biofilm is a key reason chronic wounds fail to heal [27,28]. These clinical observations provide an important benchmark against which the fidelity of 3D biofilm-containing skin models should be evaluated.

Development of 3D skin models for chronic wounds

The value of a 3D skin model for studying chronic wounds depends on how accurately it reproduces the relevant physiology. Creating architecture that mimics human skin is essential to ensure that observed outcomes reflect real wound behavior and treatment efficacy. The first and most fundamental component is a stratified epidermal layer, which provides physical integrity, innate and adaptive immune protection, and barrier homeostasis [29]. This is achieved by culturing keratinocytes at an air-liquid interface to drive differentiation of the epidermal layers, with the stratified squamous epithelium grown on an ECM embedded with dermal fibroblasts [30]. Fibroblasts, fibronectin, elastin, and collagen constitute the ECM, which provides mechanical strength and helps regulate keratinocyte and immune cell behavior; when these components become disorganized or degraded, chronic inflammation can result. Naturally derived biopolymers with flexible, hydrated, cross-linked networks help emulate the ECM and enable cell-matrix interactions critical for replicating wound pathophysiology and testing prohealing interventions [31].

The next step in complexity involves incorporating vasculature and immune components. The metabolic needs and growth of tissue depend on the surrounding vasculature, and adding this dimension makes it possible to study angiogenesis, oxygen-dependent healing, and leukocyte trafficking during chronic inflammation [17,18]. Because chronic inflammatory cascades and immune-microbe interactions are central to chronic wound pathogenesis, embedding defined immune cells, such as neutrophils, macrophages, and Langerhans cells, allows more focused study of their form and function within a near-native microenvironment [17,29]. These additions make the model more physiologically relevant and enable evaluation of therapeutic responses under more realistic conditions.

Macrophages are among the most important immune cells in chronic wound healing. This monocyte-derived cell adapts its function to environmental signals, differentiating along a spectrum whose extremes are described as M1 and M2. The M1 phenotype is proinflammatory and produces cytokines such as TNF-α and IL-1β, whereas the M2 phenotype is pro-repair and secretes factors such as IL-10 and transforming growth factor beta-1. Chronic wounds show increased M1 macrophages, which maintain a highly inflammatory state [17,18]. Neutrophils, the most abundant circulating leukocytes, act as early responders that kill pathogens using ROS, degrade debris using proteases, and secrete cytokines to control infection; however, in chronic wounds, they are excessively abundant and release elevated proteases and ROS that further damage tissue and ECM [17,18]. Incorporating these cells into 3D models helps reveal how immune dysfunction supports poor wound resolution and supports the development of targeted, proresolution treatments. For example, in a 3D collagen model of tissue repair, Sapudom et al. polarized macrophages into M2a and M2c subtypes and showed that shifting macrophage phenotype can promote fibroblast activation or resolution [32], suggesting that targeting macrophage plasticity could help reverse the immune dysfunction seen in chronic wounds.

*S. aureus*, one of the most well-known pathogens in medicine, is a common culprit in chronic wounds. Its primary mode of persistence is biofilm formation, which enables surface adherence and confers resistance to antimicrobial agents and phagocytosis [33]. It also produces toxins, including alpha-toxin and protein A. When co-cultured with immune or skin cells, *S. aureus* biofilms increase proinflammatory cytokine levels, such as IL-1β and TNF-α, slow fibroblast growth, and damage keratinocytes. *P. aeruginosa* is another key player in chronic wounds and uses quorum sensing, a cell-density-dependent signaling system, to coordinate biofilm development, virulence-factor production, and antibiotic resistance [34], operating through three central systems: Las, Rhl, and Pseudomonas quinolone signal. Keim et al. found that when *S. aureus* is coinfected with *P. aeruginosa*, it responds to *P. aeruginosa* proteases (LasA, LasB, and AprA) by forming SasG-dependent aggregates, enhancing biofilm formation, increasing antibiotic tolerance, and supporting persistence [16] (note: preprint).

Cutibacterium acnes is part of the normal skin microbiome, but in chronic wounds and on prosthetic devices, it can behave opportunistically. Like *S. aureus*, it forms resilient biofilms that contribute to persistence in chronic infection. Mechanistically, *C. acnes* modulates the immune system by engaging Toll-like receptors (TLR2 and TLR4) and triggering nuclear factor kappa B, mitogen-activated protein kinase, and NLRP3 inflammasome pathways, thereby increasing inflammation and stimulating proinflammatory cytokines including IL-1β, IL-6, IL-8, and TNF-α [35]; these effects can polarize macrophages toward a proinflammatory phenotype. It is important to place *C. acnes* in a clinical context. Compared with the most firmly established chronic wound pathogens, *S. aureus* and *P. aeruginosa*, as well as streptococci, Enterobacteriaceae, and obligate anaerobes, which together dominate cultures and 16S ribosomal RNA surveys of chronic wounds, *C. acnes* is a comparatively minor and less consistently reported wound organism. Its principal clinical relevance is in device-associated, prosthetic, and deep-tissue infections and in a subset of wound microbiomes. We therefore include *C. acnes* primarily as an instructive example of a skin commensal that can act as an immunomodulatory opportunist, rather than as a leading cause of chronic wound infection, and we have adjusted the surrounding text to reflect this relative importance. Taken together, these observations show how chronic wound organisms not only resist treatment but also actively shape the host immune environment. By incorporating key structural elements, immune cells, and wound-associated microbes, 3D skin models provide valuable insights into the interactions that drive chronic inflammation and impaired healing.

Immune-microbiome crosstalk in 3D skin models

Biofilm formation significantly modulates immune responses. Biofilms are clusters of cells within an extracellular polymeric matrix that confers resistance to antimicrobial responses and agents [36]. The matrix protects bacteria from immune cell targeting, particularly innate immune activity, as well as from antimicrobial agents [37]. Goodman et al. analyzed biofilm development in relation to the expression of matrix-associated proteins and immune suppression, and found that biofilm formation can be linked to extracellular proteins that strengthen the matrix and impede immune cell entry [38]. This physical shielding is central to chronic wound persistence because it enables bacteria to evade immunity and resist pharmacologic therapy. Similar mechanisms are frequently associated with immune evasion and the progression of chronic conditions.

Quorum sensing is a chemical signaling process by which bacteria coordinate biofilm formation. Preda and Săndulescu reported that glycolipids involved in quorum sensing disrupted neutrophil recruitment and ultimately caused necrosis, and that the absence of quorum sensing was linked with impaired biofilm development [39]. The quorum-sensing molecule N-acyl homoserine lactone, present in biofilms, induces apoptosis in macrophages [40]. These findings show that quorum sensing not only coordinates bacterial behavior but also actively disrupts host immunity. Biofilms express several virulence factors that hinder the adaptive humoral response while promoting host tissue degradation [41]. Microbial metabolites, including short-chain fatty acids, tryptophan catabolites, polyamines, and hydrogen sulfide, affect immune cell function by altering barrier function and modifying gene and enzyme expression [42]. Biofilms also contribute to oxygen depletion in their lower layers, promoting hypoxic microenvironments that favor anaerobic metabolism and reduce neutrophil recruitment [40]. These actions illustrate the strategies microbes use to shape their environment and escape immune surveillance.

Cytokines and chemokines recruit immune cells to sites of damage and play a significant role in wound healing, promoting angiogenesis and inflammatory cell migration; abnormalities can result in scarring and excessive inflammation [43]. IL-1β and TNF-α are proinflammatory cytokines central to the inflammatory response in chronic wounds [44]. Shen et al. reported that inhibiting IL-1β, and thereby blocking its downstream regulator TNF-α, improved wound healing, supporting both cytokines as therapeutic targets. Regulation of ROS is also crucial: excessive ROS and oxidative stress promote early senescence of immune cells needed for healing and contribute to chronic wound pathogenesis [45], yet the same work found that controlled amounts of hydrogen peroxide promoted healing, indicating that an optimal ROS level remains to be defined. Dysregulated immune responses are a key factor in chronic wound pathogenesis. High levels of metalloproteinases, which mediate immune cell recruitment and migration, are linked to chronic tissue degradation and impaired wound closure [46], and excessive ECM degradation further prevents closure [18]. A shift toward higher M1 and lower M2 macrophage activity increases inflammation and reduces collagen deposition [18]. High levels of neutrophil-derived proteases, such as elastase, promote neutrophil infiltration, edema, and matrix breakdown [47], whereas suppression of angiogenesis through inhibition of vascular endothelial growth factor is associated with a pathogenic phenotype and decreased neutrophil recruitment [18]. Together, these alterations show how chronic wounds derail normal immune responses and create an environment hostile to tissue regeneration.

Personalized approaches in 3D skin models

Integrating patient-derived immune cells into 3D models enables personalized modeling of wound-healing impairments and antimicrobial-resistance patterns. These models can reconstruct the structural and functional complexity of human skin, particularly the epidermis and dermis, and can mimic barrier function, cellular migration, and intercellular communication relevant to cutaneous immune responses [48]. The epidermis, composed primarily of keratinocytes, forms a physical barrier against environmental pathogens [29]; disruption of this layer activates innate immunity, and keratinocytes release proinflammatory cytokines in response to external stimuli [6,29]. CD8+ tissue-resident memory T cells reside at sites of prior infection or inflammation and can respond rapidly to recurrent pathogens [48]. The dermis contains many immune cells, including dendritic cells, macrophages, T cells, innate lymphoid cells, and mast cells, which interact with fibroblasts to coordinate immune responses through cytokines and chemokines [48]. Because cytokine and growth-factor profiles evolve throughout healing and vary between individuals, incorporating patient-derived immune cells is important for accurately modeling a unique immunological environment. The presence of bacteria attracts leukocytes, which release cytokines, proteases, and ROS from both microbial and host sources, initiating an inflammatory cascade and prolonging closure [49]. Common proinflammatory cytokines in infected skin include TNF-α, IL-8, IL-6, and IL-1α, although the specific profile varies with bacterial strain and healing stage, producing distinct cytokine cascades that influence the healing trajectory [50]. By reflecting individual variability, personalized 3D skin models enhance physiological relevance and facilitate the study of immune dysregulation and the testing of tailored interventions.

While integrating patient-derived immune cells is highly relevant to precision wound care, several technical challenges must be acknowledged. Maintaining the viability and function of primary immune cells within long-term 3D cultures is difficult, and the phenotype of macrophages, neutrophils, and T cells can drift during isolation and incubation. Reproducibility is a further concern: donor-to-donor variability, differences in cell-isolation protocols, and batch effects in scaffolds and bioinks can all reduce consistency across experiments and laboratories. Sustaining a stable, physiologically representative microbiome over the culture period is also challenging because fast-growing organisms may overtake the community, biofilm maturation is time-dependent, and antibiotic-free conditions increase the risk of overgrowth. Despite these hurdles, personalized models have concrete clinical potential. For diagnostics, patient-specific constructs could help characterize an individual’s inflammatory phenotype and biofilm susceptibility. For therapeutic testing, they offer a controlled setting in which to compare anti-biofilm, immune-modulating, and microbiome-targeted strategies before clinical use. For risk stratification, differences in immune and microbial responses between patient-derived models may eventually help identify individuals at higher risk of delayed healing or antimicrobial resistance. Realizing these applications will require standardized protocols, validated readouts, and prospective comparison with clinical outcomes.

One major advantage of personalized 3D models is the ability to capture individual variation in wound healing and antimicrobial resistance. Chronic wounds, including vascular, pressure, and diabetic ulcers, are characterized by disrupted repair and persistent microbial colonization, and impaired healing is often associated with sustained inflammation, hypoxia, ischemia, recurrent infection, and biofilm formation [51]. Partial- and full-thickness wounds, particularly those larger than 4 cm, are associated with prolonged healing, motivating development of multifunctional bioscaffolds that support regeneration in deep and chronic wounds by enhancing cell contact, helping to prevent infection, and degrading at a controlled rate; however, their efficacy may vary among patients according to wound type, depth, and immune profile, reinforcing the need for personalized modeling [52]. To investigate a patient-specific microenvironment, advanced 3D skin models can integrate skin layers, patient-derived cell lines, immune cells, vascular-like networks, and the patient’s microbiota. Despite this potential, integrating patient-derived cells, especially adaptive immune cells, remains a challenge and a critical factor in studying the drivers of impaired healing [51]. Modeling antimicrobial resistance in these settings may offer new insights into microbial persistence and immune variation.

Three-dimensional skin models have been developed in which bacterial strains are inoculated into the epidermis, allowing assessment of epidermal barrier integrity, bacterial penetration, and drug efficacy against pathogens, particularly in the context of biofilm. In one study, *S. aureus *and *Escherichia coli* were independently introduced into the epidermis, with sampling at 24 and 48 hours; bacterial proliferation occurred without penetration into the dermis, indicating barrier preservation. A complementary wounded model inoculated with *S. aureus* and *E. coli* was used to assess penicillin-streptomycin treatment, which reduced colonization and preserved barrier integrity, demonstrating drug efficacy [50]. Incorporating patient-derived cells allows these models to simulate individual immune responses and to reveal how immune profiles influence microbial susceptibility and drug effectiveness. With appropriate validation, such personalization may help predict clinical outcomes and inform targeted treatment strategies, although this remains an aspiration rather than an established capability.

The development of 3D skin models shows promise for precision medicine in chronic wound management. By creating a personalized model reflecting a patient’s immune and microbial profile, researchers can study therapeutic responses in a controlled setting. Several challenges remain, however, in reproducing the structural and immunological complexity of a specific donor. Many RHE models have limited lifespans, complicating the study of slow-growing pathogens and long-term microbiome stability, and RHE models often lack secondary skin structures and incur relatively high production costs [53]. An important aspect of personalized modeling is integrating patient-specific immune cells alongside faithful reproduction of donor skin composition. One of the most impactful applications is the potential to predict individual biological variation, including inflammatory responses that differ among patients due to disease phenotype and drug response [48]. Although some 3D skin models have incorporated proinflammatory cytokines such as TNF-α, IL-8, IL-6, and IL-1α, many lack vascular integration, which limits their relevance to precision medicine [50]. Even so, such models have demonstrated variable cytokine expression across bacterial strains, and further incorporation of vasculature and immune components is expected to improve predictivity. Ultimately, faithful integration of patient-specific immunological features may reveal new mechanisms of disease progression and support more effective, personalized therapies, with the potential to improve outcomes and quality of life for patients with chronic wounds.

Applications of 3D skin models

Because 3D skin models reproduce tissue architecture and immune-microbe interactions more faithfully than 2D cultures, they support a range of applications relevant to chronic wound care. For clarity, we organize these applications into four areas: antibiofilm and drug testing, immune modulation, microbiome-targeted therapies, and precision medicine. A synthesis of the individual model studies discussed throughout this review is provided in Table 2, and representative therapeutic applications are summarized in Table 3.

**Table 2:** Synthesis of representative 3D (and comparative 2D) skin/wound model studies discussed in this review 2D: two-dimensional; 3D: three-dimensional; CCR6: chemokine receptor 6; CLA: cutaneous lymphocyte antigen; PDE4i: phosphodiesterase 4 inhibitors; IL: interleukin; AMP: antimicrobial peptide

Study	Model type	Cellular/immune components	Microbial species	Main outcome	Key limitation
Chen et al. [10]	2D vs. 3D epidermal model	Keratinocytes	None	3D model avoided cytotoxicity/oxidative stress seen in 2D at equal dose	Epidermis only; no immune/microbe
Loomis et al. [14]	3D skin model	Keratinocytes/fibroblasts	Mixed skin microbiome	Microbiome altered epidermal thickness, gene expression, proliferation	Avascular; limited immune complexity
Sapudom et al. [32]	3D collagen tissue-repair	Macrophages, fibroblasts	None	M2a/M2c polarization drives fibroblast activation/resolution	No microbial component; avascular
Keim et al. [16] (preprint)	Polymicrobial biofilm model	Bacterial co-culture	*S. aureus* + *P. aeruginosa*	Protease-driven SasG aggregation boosts biofilm/tolerance	Preprint; not a full skin construct
Shin et al. [57]	Immunocompetent skin construct	Th1/Th17, CCR6+CLA+ T cells, epidermis	None	T-cell migration; drug response (PDE4i, anti-IL-17A)	Avascular; no microbial component
Lemoine et al. [58]	Microbially competent 3D skin	Keratinocytes/fibroblasts	*M. luteus*, *P. oleovorans*	Microbial diversity shapes immune modulation and AMPs	Commensal, not classic wound pathogens
Villata et al. [50]	3D epidermal/wounded model	Keratinocytes (+cytokines)	*S. aureus*, *E. coli*	Barrier assessment; penicillin-streptomycin efficacy	Avascular; limited immune complexity
Ali et al. [56]	3D biofilm model	3D skin construct	S. aureus	Combination antibiotics eradicate biofilm	Single-strain; limited immune complexity

**Table 3:** Representative therapeutic applications of 3D skin models in chronic wound research 2D: two-dimensional; 3D: three-dimensional; IL-17A: interleukin 17A; IFN-γ: interferon gamma; TNF-α: tumor necrosis factor alpha; AMP: antimicrobial peptide; PDE4: phosphodiesterase 4

Application area	Example approach/model	Key finding or rationale	Reference
Antibiofilm/drug testing	3D skin model with *S. aureus* biofilm	Bacitracin + chlorhexidine digluconate eradicated biofilm better than monotherapy	[56]
Immune modulation	Immunocompetent construct with Th1/Th17 and patient-derived T cells	T-cell migration and IL-17A/IFN-γ/TNF-α output; responded to PDE4 inhibitors and anti-IL-17A	[57]
Microbiome-targeted therapy	3D skin with commensal *M. luteus* and *P. oleovorans*	Microbial diversity shapes immune modulation, AMP production, and growth factors	[58]
Barrier/infection modeling	Wounded 3D epidermal model inoculated with *S. aureus* and *E. coli*	Penicillin-streptomycin reduced colonization and preserved the barrier	[50]
Macrophage-directed repair	3D collagen tissue-repair model	M2a/M2c polarization promotes fibroblast activation and resolution	[32]
Precision medicine	Patient-derived cells + microbiota in advanced constructs	Personalized modeling of immune/microbial variation (preclinical)	[48,51]

Antibiofilm and drug testing

Biofilm-producing bacteria, including *S. aureus*, *P. aeruginosa*, and *C. acnes*, frequently colonize chronic wounds, and their biofilms defend against both host immunity and antibiotics [54]. Traditional 2D models lack the spatial architecture needed to replicate human skin, making 3D models more physiologically relevant for this purpose [55]. These platforms have proven useful for testing antibiofilm compounds; for example, using 3D skin models, Ali et al. found that combining bacitracin and chlorhexidine digluconate eradicated *S. aureus* biofilms more effectively than either monotherapy [56]. Because chronic inflammation can compromise repair, therapeutics that also regulate inflammatory pathways, in addition to eliminating infection, are likely to be needed.

Immune modulation

Nonhealing wounds, including pressure, diabetic, and venous ulcers, are characterized by persistent inflammation and elevated proinflammatory cytokines and ROS, which degrade the ECM, impair migration, and stall the proliferative phase [49]. Integrating immune cells into 3D models helps replicate immune responses and study signaling pathways, cytokine cascades, and cell recruitment. Shin et al. developed immunocompetent human skin constructs using Th1/Th17 cells and patient-derived chemokine receptor 6 (CCR6) + cutaneous lymphocyte antigen + T cells [57]; when the epidermis was present, T cells migrated, sustained activation, and increased production of IL-17A, interferon gamma, and TNF-α, and the constructs responded to therapeutics such as hydrocortisone, phosphodiesterase-4 inhibitors, and anti-IL-17A antibodies, validating the platform for testing immune-modulating therapies under disease-relevant conditions. Incorporating patient-derived immune cells could further tailor efficacy to an individual response.

Microbiome-targeted therapies

Microbiome dysregulation can drive chronic wound persistence by impairing epithelial repair and sustaining inflammation. Incorporating patient-derived microbiomes enables evaluation of host-bacterial interactions, metabolite signaling, and quorum sensing. Using commensal strains of *Micrococcus luteus* and *Pseudomonas oleovorans*, Lemoine et al. studied immune modulation, antimicrobial peptide production, and growth factor expression in a 3D skin model [58], finding that microbial diversity shapes local immune responses and suggesting that these models are well suited to evaluating personalized interventions. Strategies such as prebiotics and probiotics may help restore dysregulated microbial communities and reduce inflammation.

Precision medicine and regulatory context

Advances in 3D skin models are enabling a shift from preclinical toward clinical, real-world applications. The regulatory landscape is also evolving, but its implications should be stated precisely. The U.S. Food and Drug Administration (FDA) Modernization Act 2.0 (2022) amended the Federal Food, Drug, and Cosmetic Act to permit, but not require, the use of validated nonanimal new approach methodologies (NAMs), such as cell-based assays, microphysiological systems, and bioprinted models, in support of drug applications. Importantly, the Act removed the previous blanket requirement for animal testing rather than establishing 3D skin models as automatic or qualified substitutes for animal studies; regulatory acceptance of any specific method still depends on its context of use and on formal validation. More recent FDA activity, including initiatives to reduce reliance on animal testing, signals growing support for NAMs but does not guarantee their acceptance in all drug-development contexts [57]. Within this framework, incorporating a broader array of immune cells makes the modeled microenvironment more representative of human skin, and 3D bioprinting can fabricate full-thickness skin incorporating neurovasculature and hair follicles to improve functionality and integrity [59]. Skin-on-a-chip systems emulate blood-flow environments, cell migration, and mechanical forces and are particularly useful for modeling systemic disease responses and complex drug-delivery dynamics. Together, these advances provide a predictive platform for drug screening and disease modeling that can help reduce reliance on animal models [57].

Limitations

Despite their considerable promise, 3D skin models for studying chronic wounds face several important limitations, which we group into biological, technical, and translational categories. Biologically, many RHE models have a limited lifespan, which hinders investigation of slow-growing pathogens and long-term microbiome stability, and most current models lack vascular integration, restricting the study of oxygen-dependent healing, leukocyte trafficking, and systemic inflammatory responses. Incorporating full immune complexity remains technically challenging, particularly the inclusion of patient-derived adaptive immune cells alongside innate components. The absence of secondary skin structures such as hair follicles, sebaceous glands, and sweat glands reduces physiological relevance. High production costs associated with RHE models may limit broader adoption.

A further and often underappreciated set of limitations concerns fidelity to real wound microbiology and cross-study comparability. Many studies rely on single-strain inoculations, whereas chronic wounds are typically polymicrobial; single-strain models therefore underrepresent the interspecies competition, cooperation, and metabolic exchange that shape clinical biofilms. Maintaining mature, stable polymicrobial biofilms in vitro is difficult because faster growing organisms can dominate, and biofilm architecture is sensitive to culture conditions. The immune compartment is also incompletely represented in most models, which capture selected innate populations but rarely the full innate-adaptive repertoire, its spatial organization, or its temporal dynamics. Compounding these issues, there is at present no widely adopted standardization of 3D wound-model protocols, which limits reproducibility between laboratories, and few models have been formally validated against human chronic wound samples. As a result, a substantial translational gap persists between short-term in vitro findings and clinical wound-healing outcomes. We regard acknowledgment of these gaps as constructive rather than undermining, because it identifies precisely the standardization, validation, and benchmarking work the field still needs. Addressing these limitations will be critical to advancing 3D skin models as reliable platforms for precision medicine in chronic wound management.

Future perspectives

Several converging technologies are likely to shape the next generation of 3D skin models. First, artificial intelligence and machine learning are expected to assist model optimization, from designing bioink formulations and print parameters to automating image-based quantification of biofilm, immune infiltration, and reepithelialization, and to predicting therapeutic responses from complex, high-content datasets. Second, multiomics integration, combining transcriptomics, proteomics, metabolomics, and microbiome sequencing, will help link molecular signatures across these constructs to wound phenotypes and treatment responses and support the identification of biomarkers for risk stratification. Third, vascularized skin-on-a-chip systems that incorporate perfusable microvasculature, controllable oxygen gradients, and immune cell recruitment will more faithfully reproduce the ischemic, hypoxic, and inflammatory conditions of chronic wounds and enable dynamic, longitudinal study of host-microbe interactions. Fourth, advances in large-scale, standardized manufacturing, including automated bioprinting and quality-controlled production of reproducible constructs, will be essential to move these platforms from bespoke laboratory tools toward robust, comparable assays suitable for drug development and, eventually, clinical decision support.

Realizing this potential will require deliberate progress on standardization, cross-laboratory validation, and prospective benchmarking against human chronic wound data. If these steps are achieved, vascularized, immunocompetent, and microbiome-competent 3D skin models could become integral to translational wound care, supporting patient-specific selection of antibiofilm, immune-modulating, and microbiome-targeted therapies and thereby advancing genuinely personalized medicine for chronic wounds.

## Conclusions

Three-dimensional skin models are becoming an important tool for studying chronic wounds because they emulate human skin more closely than traditional 2D cultures. By integrating immune cells, ECM components, and key wound-associated microbes such as *S. aureus*, *P. aeruginosa*, and *C. acnes*, these models allow investigation of the relationships between inflammation, biofilm formation, and impaired healing. They have shown promise for studying immune dysregulation, cytokine signaling, and therapeutic responses, and for supporting more targeted, personalized approaches to wound care. At the same time, the current evidence remains predominantly preclinical, and challenges such as limited vascularization, short culture lifespan, incomplete immune representation, difficulty modeling polymicrobial biofilms, and lack of standardization must be addressed. Looking ahead, the integration of vascularized skin-on-a-chip platforms, multiomics readouts, AI-assisted optimization, and standardized manufacturing offers a credible path from proof-of-concept models toward validated, clinically actionable tools. With rigorous validation against human wound data, these models have real potential to translate into clinical practice and to enable more effective, personalized treatment for the millions of people living with chronic wounds.

## References

[1] Darwin E, Tomic-Canic M (2018). Healing chronic wounds: current challenges and potential solutions. Curr Dermatol Rep.

[2] Frykberg RG, Banks J (2015). Challenges in the treatment of chronic wounds. Adv Wound Care (New Rochelle).

[3] Sen CK (2023). Human wound and its burden: updated 2022 compendium of estimates. Adv Wound Care (New Rochelle).

[4] Armstrong DG, Swerdlow MA, Armstrong AA, Conte MS, Padula WV, Bus SA (2020). Five year mortality and direct costs of care for people with diabetic foot complications are comparable to cancer. J Foot Ankle Res.

[5] Díaz GY, da Silva VA, Kalantarnia F (2025). Using three-dimensional bioprinting to generate realistic models of wound healing. Adv Wound Care (New Rochelle).

[6] Smythe P, Wilkinson HN (2023). The skin microbiome: current landscape and future opportunities. Int J Mol Sci.

[7] Williams MR, Nakatsuji T, Sanford JA, Vrbanac AF, Gallo RL (2017). Staphylococcus aureus induces increased serine protease activity in keratinocytes. J Invest Dermatol.

[8] Rademacher F, Simanski M, Hesse B, Dombrowsky G, Vent N, Gläser R, Harder J (2019). Staphylococcus epidermidis activates aryl hydrocarbon receptor signaling in human keratinocytes: implications for cutaneous defense. J Innate Immun.

[9] Galvan A, Pellicciari C, Calderan L (2024). Recreating human skin in vitro: should the microbiota be taken into account?. Int J Mol Sci.

[10] Chen L, Wu M, Jiang S (2019). Skin toxicity assessment of silver nanoparticles in a 3D epidermal model compared to 2D keratinocytes. Int J Nanomedicine.

[11] Clément V, Roy V, Paré B (2022). Tridimensional cell culture of dermal fibroblasts promotes exosome-mediated secretion of extracellular matrix proteins. Sci Rep.

[12] Gao C, Lu C, Jian Z (2021). 3D bioprinting for fabricating artificial skin tissue. Colloids Surf B Biointerfaces.

[13] Tan SH, Ngo ZH, Sci DB, Leavesley D, Liang K (2022). Recent advances in the design of three-dimensional and bioprinted scaffolds for full-thickness wound healing. Tissue Eng Part B Rev.

[14] Loomis KH, Wu SK, Ernlund A, Zudock K, Reno A, Blount K, Karig DK (2021). A mixed community of skin microbiome representatives influences cutaneous processes more than individual members. Microbiome.

[15] Baethge C, Goldbeck-Wood S, Mertens S (2019). SANRA-a scale for the quality assessment of narrative review articles. Res Integr Peer Rev.

[16] Keim K, Bhattacharya M, Crosby HA, Jenul C, Mills K, Schurr M, Horswill A (2024). Polymicrobial interactions between Staphylococcus aureus and Pseudomonas aeruginosa promote biofilm formation and persistence in chronic wound infections. [Preprint]. bioRxiv.

[17] Raziyeva K, Kim Y, Zharkinbekov Z, Kassymbek K, Jimi S, Saparov A (2021). Immunology of acute and chronic wound healing. Biomolecules.

[18] Versey Z, da Cruz Nizer WS, Russell E (2021). Biofilm-innate immune interface: contribution to chronic wound formation. Front Immunol.

[19] Uberoi A, McCready-Vangi A, Grice EA (2024). The wound microbiota: microbial mechanisms of impaired wound healing and infection. Nat Rev Microbiol.

[20] Tang Q, Xue N, Ding X (2023). Role of wound microbiome, strategies of microbiota delivery system and clinical management. Adv Drug Deliv Rev.

[21] Mihai MM, Bălăceanu-Gurău B, Ion A (2024). Host-microbiome crosstalk in chronic wound healing. Int J Mol Sci.

[22] Kalan LR, Brennan MB (2019). The role of the microbiome in nonhealing diabetic wounds. Ann N Y Acad Sci.

[23] Senneville É, Albalawi Z, van Asten SA (2023). IWGDF/IDSA guidelines on the diagnosis and treatment of diabetes-related foot infections (IWGDF/IDSA 2023). Clin Infect Dis.

[24] Alam W, Hasson J, Reed M (2021). Clinical approach to chronic wound management in older adults. J Am Geriatr Soc.

[25] Beyene RT, Derryberry SL Jr, Barbul A (2020). The effect of comorbidities on wound healing. Surg Clin North Am.

[26] Maciel-Fiuza MF, Muller GC, Campos DM (2023). Role of gut microbiota in infectious and inflammatory diseases. Front Microbiol.

[27] Malone M, Bjarnsholt T, McBain AJ (2017). The prevalence of biofilms in chronic wounds: a systematic review and meta-analysis of published data. J Wound Care.

[28] Bjarnsholt T, Kirketerp-Møller K, Jensen PØ (2008). Why chronic wounds will not heal: a novel hypothesis. Wound Repair Regen.

[29] Baroni A, Buommino E, De Gregorio V, Ruocco E, Ruocco V, Wolf R (2012). Structure and function of the epidermis related to barrier properties. Clin Dermatol.

[30] Carlson MW, Alt-Holland A, Egles C, Garlick JA (2008). Three-dimensional tissue models of normal and diseased skin. Curr Protoc Cell Biol.

[31] Tan SH, Chua DA, Tang JR, Bonnard C, Leavesley D, Liang K (2022). Design of hydrogel-based scaffolds for in vitro three-dimensional human skin model reconstruction. Acta Biomater.

[32] Sapudom J, Karaman S, Mohamed WK, Garcia-Sabaté A, Quartey BC, Teo JC (2021). 3D in vitro M2 macrophage model to mimic modulation of tissue repair. NPJ Regen Med.

[33] Peng Q, Tang X, Dong W, Sun N, Yuan W (2022). A review of biofilm formation of Staphylococcus aureus and its regulation mechanism. Antibiotics (Basel).

[34] Miranda SW, Asfahl KL, Dandekar AA, Greenberg EP (2022). Pseudomonas aeruginosa quorum sensing. Adv Exp Med Biol.

[35] Mayslich C, Grange PA, Dupin N (2021). Cutibacterium acnes as an opportunistic pathogen: an update of its virulence-associated factors. Microorganisms.

[36] Kiedrowski MR, Kavanaugh JS, Malone CL (2011). Nuclease modulates biofilm formation in community-associated methicillin-resistant Staphylococcus aureus. PLoS One.

[37] Kostakioti M, Hadjifrangiskou M, Hultgren SJ (2013). Bacterial biofilms: development, dispersal, and therapeutic strategies in the dawn of the postantibiotic era. Cold Spring Harb Perspect Med.

[38] Goodman SD, Obergfell KP, Jurcisek JA (2011). Biofilms can be dispersed by focusing the immune system on a common family of bacterial nucleoid-associated proteins. Mucosal Immunol.

[39] Preda VG, Săndulescu O (2019). Communication is the key: biofilms, quorum sensing, formation and prevention. Discoveries (Craiova).

[40] Mirzaei R, Sabokroo N, Ahmadyousefi Y, Motamedi H, Karampoor S (2022). Immunometabolism in biofilm infection: lessons from cancer. Mol Med.

[41] Cangui-Panchi SP, Ñacato-Toapanta AL, Enríquez-Martínez LJ, Salinas-Delgado GA, Reyes J, Garzon-Chavez D, Machado A (2023). Battle royale: immune response on biofilms - host-pathogen interactions. Curr Res Immunol.

[42] Liu J, Tian R, Sun C, Guo Y, Dong L, Li Y, Song X (2023). Microbial metabolites are involved in tumorigenesis and development by regulating immune responses. Front Immunol.

[43] Ridiandries A, Tan JT, Bursill CA (2018). The role of chemokines in wound healing. Int J Mol Sci.

[44] Shen S, Miskolci V, Dewey CN, Sauer JD, Huttenlocher A (2024). Infection induced inflammation impairs wound healing through IL-1β signaling. iScience.

[45] Hunt M, Torres M, Bachar-Wikstrom E, Wikstrom JD (2024). Cellular and molecular roles of reactive oxygen species in wound healing. Commun Biol.

[46] Wysocki AB, Staiano-Coico L, Grinnell F (1993). Wound fluid from chronic leg ulcers contains elevated levels of metalloproteinases MMP-2 and MMP-9. J Invest Dermatol.

[47] Diegelmann RF (2003). Excessive neutrophils characterize chronic pressure ulcers. Wound Repair Regen.

[48] Moon S, Kim DH, Shin JU (2021). In vitro models mimicking immune response in the skin. Yonsei Med J.

[49] Zhao R, Liang H, Clarke E, Jackson C, Xue M (2016). Inflammation in chronic wounds. Int J Mol Sci.

[50] Villata S, Baruffaldi D, Cue Lopez R (2024). Broadly accessible 3D in vitro skin model as a comprehensive platform for antibacterial therapy screening. ACS Appl Mater Interfaces.

[51] Hofmann E, Fink J, Pignet AL (2023). Human in vitro skin models for wound healing and wound healing disorders. Biomedicines.

[52] Fadilah NI, Riha SM, Mazlan Z (2023). Functionalised-biomatrix for wound healing and cutaneous regeneration: future impactful medical products in clinical translation and precision medicine. Front Bioeng Biotechnol.

[53] Larson PJ, Chong D, Fleming E, Oh J (2021). Challenges in developing a human model system for skin microbiome research. J Invest Dermatol.

[54] Sharma S, Mohler J, Mahajan SD, Schwartz SA, Bruggemann L, Aalinkeel R (2023). Microbial biofilm: a review on formation, infection, antibiotic resistance, control measures, and innovative treatment. Microorganisms.

[55] Cometta S, Hutmacher DW, Chai L (2024). In vitro models for studying implant-associated biofilms - a review from the perspective of bioengineering 3D microenvironments. Biomaterials.

[56] Ali HR, Collier P, Bayston R (2024). A three-dimensional model of bacterial biofilms and its use in antimicrobial susceptibility testing. Microorganisms.

[57] Shin JU, Abaci HE, Herron L (2020). Recapitulating T cell infiltration in 3D psoriatic skin models for patient-specific drug testing. Sci Rep.

[58] Lemoine L, Dieckmann R, Al Dahouk S, Vincze S, Luch A, Tralau T (2020). Microbially competent 3D skin: a test system that reveals insight into host-microbe interactions and their potential toxicological impact. Arch Toxicol.

[59] Yang K, Wang L, Vijayavenkataraman S, Yuan Y, Tan EC, Kang L (2024). Recent applications of three-dimensional bioprinting in drug discovery and development. Adv Drug Deliv Rev.

